# Identification of a novel lncRNA prognostic signature and analysis of functional lncRNA AC115619.1 in hepatocellular carcinoma

**DOI:** 10.3389/fphar.2023.1167418

**Published:** 2023-08-08

**Authors:** Binliang Gan, Youwu He, Yonggang Ma, Linfeng Mao, Chuanjie Liao, Ganlu Deng

**Affiliations:** ^1^ Department of Oncology, The First Affiliated Hospital of Guangxi Medical University, Nanning, Guangxi, China; ^2^ Department of Oncology, The Fifth Affiliated Hospital of Guangxi Medical University, Nanning, Guangxi, China; ^3^ Key Laboratory of Early Prevention and Treatment for Regional High Frequency Tumor (Guangxi Medical University), Ministry of Education, Nanning, Guangxi, China; ^4^ Department of NeuroInterventional Surgery, Binzhou Medical University Hospital, Binzhou, Shandong, China; ^5^ Department of Hepatobiliary Surgery, The First Affiliated Hospital of Guangxi Medical University, Nanning, Guangxi, China

**Keywords:** long non-coding RNA, hepatocellular carcinoma, biomarker, diagnosis, prognosis

## Abstract

**Background:** Hepatocellular carcinoma (HCC) is the deadliest malignancy. Long non-coding RNAs (lncRNAs) are involved in the development of multiple human malignancies. This study aimed to establish a reliable signature and identify novel biomarkers for HCC patients.

**Methods:** Differentially expressed lncRNAs (DElncRNAs) were identified from Gene Expression Omnibus (GEO) and The Cancer Genome Atlas (TCGA) databases. Univariate, LASSO, and multivariate Cox regression analyses were applied to screen the prognostic lncRNAs and establish a prognostic model. Receiver operating characteristic (ROC) curves and Kaplan–Meier analyses were conducted to validate the prognostic value of this model. The association between lncRNAs and differential m6A genes was analyzed by Spearman’s analysis. A series of bioinformatic and *in vitro* experiments were applied to explore the function of hub lncRNA.

**Results:** A total of 32 DElncRNAs were identified, and 12 DElncRNAs were associated with the prognosis of HCC patients. A prognostic signature comprising six prognostic lncRNAs (LINC02428, LINC02163, AC008549.1, AC115619.1, CASC9, and LINC02362) was constructed, and the model exhibited an excellent capacity for prognosis prediction. Furthermore, 12 differential m6A regulators were identified, and RBMX was found to be correlated negatively with the hub lncRNA AC115619.1. The expression level of AC115619.1 was lower in HCC tissues than that in normal tissues and was significantly related to clinicopathologic features, survival rate, and drug sensitivity. Overexpression of AC115619.1 notably inhibited the proliferation, migration, and invasion of HCC cells.

**Conclusion:** This study provided a promising prognostic signature for HCC patients and identified AC115619.1 as a novel biomarker, which plays an essential role in regulating the progression of HCC.

## 1 Introduction

Primary liver cancer is one of the most common malignancies worldwide, with about 906,000 new cases and 830,000 deaths reported in 2020. Hepatocellular carcinoma (HCC) accounts for the majority of incidence and mortality with a 75%–85% constitution statistically (Sung et al., 2021). Despite the great efforts and huge improvement in diagnosis and treatment therapies, the prognosis of HCC is still poor with a 5-year survival rate of approximately 12% (Petrowsky et al., 2020). The poor outcome of HCC poses a tremendous burden to social economy and public health. With the obscure symptom of early-stage HCC, most HCC patients are diagnosed at a late stage and lose their opportunity to receive radical resection (Anwanwan et al., 2020; Demir et al., 2021). In addition, recurrence, metastasis, and chemoresistance present major barriers to a satisfactory effect for HCC treatment (Kim et al., 2017). Therefore, highly efficient and specific biomarkers are still needed for diagnosis and prognostic prediction, which could improve the poor prognosis and individualized treatments for HCC patients.

Accumulating evidence has demonstrated that long non-coding RNA (lncRNA), which is defined as RNA transcripts of more than 200 base pairs in length (Esteller, 2011), has contributed to tumorigenesis, progression, and metastasis (Schmitt and Chang, 2016; Calle et al., 2018). LncRNAs exert various biological effects to participate in the biological and pathological processes by regulating multiple processes including transcription, epigenesis, and mRNA expression (Wang and Chang, 2011; Dykes and Emanueli, 2017; Ransohoff et al., 2018; Herman et al., 2022). The aberrant expression of lncRNA has been identified as “oncogenes” or “tumor suppressors,” as well as a prognostic factor of cancer patients. Several lncRNAs have been identified to play a role in HCC. For example, lncRNAs MALAT1, PVT1, and HOTAIR have been found to contribute to the prognosis and different cellular phenotypes such as proliferation and metastasis in human malignancies (Zhao et al., 2018; Rajagopal et al., 2020; Shigeyasu et al., 2020; Goyal et al., 2021). Ni et al. proposed that lncRNA uc.134 was downregulated in HCC and directly conferred with the patient’s prognosis. LncRNA uc.134 inhibited proliferation and metastasis by suppressing CUL4A-mediated ubiquitination of LATS1 (Ni et al., 2017).

Although many previous studies focused on the functions of lncRNAs, exploring a novel biomarker of lncRNA is still needed. Given the promising role of lncRNAs in HCC, we aimed to identify a lncRNA-related prognosis biomarker and elucidate its function in HCC. In this work, we identified lncRNAs expressed differentially in multiple public databases and constructed a prognostic prediction model by bioinformatics analysis. Systematic analysis showed that lncRNA AC115619.1 contributed excellent value in patients’ survival, but its function has never been explored previously. A ceRNA network and functional enrichment of lncRNA AC115619.1 were also applied, as well as analysis of drug sensitivity. Additionally, we verified the downregulation of AC115619.1 in local HCC samples. Functional experiments revealed that overexpression of AC115619.1 inhibited the progression of HCC. Our study might develop a novel biomarker and provide more insights to better understand the molecular mechanism of HCC.

## 2 Materials and methods

### 2.1 Data acquisition

The expression level of the lncRNA microarray was obtained from the Gene Expression Omnibus (GEO) database. Studies were chosen from GEO according to the following criteria: 1) studies with HCC tissue and adjacent normal tissue samples; 2) studies with information on the technology and platform utilized for studies. Based on these criteria, nine microarray datasets (GSE138178, GSE93789, GSE101728, GSE115018, GSE70880, GSE67260, GSE58043, GSE55191, and GSE84004) were downloaded from the GEO repository. Details of each microarray study are provided in Table 1. Meanwhile, the RNA sequencing data (374 tumor samples and 50 normal liver samples; type: FPKM), and the corresponding clinical and prognostic information on HCC patients were obtained from The Cancer Genome Atlas (TCGA) (https://portal.gdc.cancer.gov/) database. Samples with gene expression of “0” value and insufficient clinical and survival information were excluded.

**TABLE 1:** Details of the lncRNA microarray from the GEO database.

GEO ID	Platform	Sample	Numbers (tumor)	Numbers (normal)
GSE138178	GPL21827	HCC	49	49
GSE93789	GPL16956	HCC	5	5
GSE101728	GPL21047	HCC	7	7
GSE115018	GPL20115	HCC	12	12
GSE70880	GPL19748	HCC	16	16
GSE67260	GPL19072	HCC	5	5
GSE58043	GPL13825	HCC	7	7
GSE55191	GPL15314	HCC	3	3
GSE84004	GPL22109	HCC	38	38

### 2.2 Reannotation of microarray probes

LncRNA expression profiles were downloaded from the GEO database with probe ID and sequences. A custom pipeline was performed to re-annotate the probes of the lncRNA microarray. The corresponding sequences of the re-annotated probes were uniquely mapped to the human genome with no mismatch, and the chromosomal position of the retained probes was subsequently matched to the chromosomal position of lncRNAs or protein-coding genes from the GENCODE project (https://www.gencodegenes.org).

### 2.3 Identification of robust DElncRNAs

Batch normalization and the R package “limma” were utilized to generate differentially expressed lncRNAs. The aforementioned nine GSE datasets were then integrated and filtrated using robust rank aggregation (RRA). eBayes was used for identifying DElncRNAs in HCC samples compared with adjacent normal tissues with the criteria of |log_2_FC| > 1, adjusted *p* < 0.05. The dysregulated lncRNA lists from the GEO and TCGA platforms were converged for further analysis.

### 2.4 Construction of a prognostic model

Corresponding survival information on HCC patients was obtained from TCGA dataset. To filter the potential prognostic lncRNAs in HCC patients, we performed univariate regression analysis and subsequent least absolute shrinkage and selection operator (LASSO) regression to carry out prognostic analysis. Multivariate Cox regression analyses were then used to determine which lncRNA was an independent prognostic factor for HCC patients. All HCC patients from TCGA dataset were randomly divided into training and test cohorts for further prognostic prediction for lncRNAs. Both training and test cohorts were then implemented with a risk score model calculated with the formula, risk score = $\sum_{1}^{6}coefficient\;of\;lncRNA*expression\;of\;lncRNA$ . LncRNA represented the six lncRNAs screened from multivariate Cox regression. Kaplan–Meier analysis and the log-rank test were applied to compare the low- and high-risk subgroups and additional subgroups based on the median values of the risk score. A receiver operating characteristic (ROC) analysis was performed to estimate the value of the prognostic model. Finally, univariate and multivariate Cox regression analyses were applied to evaluate whether the risk score was an independent prognostic factor when combined with other clinical characteristics.

### 2.5 Association analysis between prognostic lncRNAs and m6A regulators

The expression profile of m6A-related regulators was obtained from TCGA database, as well as the corresponding survival information on HCC patients. Then, the univariate Cox regression analysis was applied to estimate the prognostic value of m6A-related regulators using the “survival” R package. Pearson’s correlation analysis was subsequently implemented to investigate the correlation of HCC prognostic-related lncRNAs and m6A-related regulators.

### 2.6 Validation of the expression and evaluation of the clinical significance of lncRNA AC115619.1 for HCC patients in public databases

To further validate the expression of the identified hub lncRNA AC115619.1 in HCC tumor tissues compared to adjacent normal tissues, the expression profiles of several GSE datasets were downloaded and analyzed. The clinicopathologic and prognostic information on patients from TCGA database was then used to determine the clinical significance of lncRNA AC115619.1. The differences among the clinicopathologic factors, including tumor grade, tumor invasion, and TNM stage, were evaluated. Kaplan–Meier survival curve analysis was conducted to demonstrate the overall survival (OS) of patients with different expression levels of AC115619.1 using the survival R package.

### 2.7 CeRNA regulatory network and functional enrichment analysis

A ceRNA network was constructed to explore the regulatory relationship. The miRcode database (http://www.mircode.org/) was applied to predict the target miRNAs of AC115619.1. Potential target mRNAs of the miRNAs were then screened using miRDB (http://www.mirdb. org/), miRTarBase (https://mirtarbase.cuhk.edu.cn/), and TargetScan databases together. To acquire more accurate target mRNAs, target mRNAs were subsequently filtered by intersecting with HCC-related differentially expressed genes from TCGA database (|log_2_FC| > 2, *p* < 0.01). The lncRNA–miRNA–mRNA ceRNA network was visualized using Cytoscape software. Gene Ontology (GO) analysis and Kyoto Encyclopedia of Genes and Genomes (KEGG) enrichment analysis were performed to investigate the functions and potential signaling pathways of differentially expressed lncRNAs using the R package “clusterProfiler.” GO includes the following three major groups: biological processes, cellular components, and molecular functions.

### 2.8 Estimation of immunocyte infiltration and analysis of drug sensitivity

The CIBERSORT algorithm was applied to estimate the proportion of immune cell infiltration in 22 human hematopoietic cell phenotypes between high- and low-AC115619.1 groups. To improve the clinical application of AC115619.1, the pRRophetic (https://github.com/paulgeeleher/pRRophetic) R package was used to predict the sensitivity of chemotherapeutic and targeted agents between high- and low-AC115619.1 patients. The half-maximal inhibitory concentration (IC_50_) of the targeted and chemotherapeutic agents for each patient were predicted using the R package based on the pretreated gene expression and drug sensitivity data on cancer cell lines.

### 2.9 Tissue collection

A total of 43 tumor tissue samples and paired adjacent normal tissue samples were randomly collected from HCC patients who were admitted to The First Affiliated Hospital of Guangxi Medical University. All specimens were routinely processed for a pathological diagnosis of surgeries according to the WHO classification. No patients received radiotherapy, chemotherapy, or immunotherapy before the samples were collected. The study was approved by the Research Ethics Committee of Guangxi Medical University. Informed consent was obtained from all participating patients.

### 2.10 Quantitative real-time reverse transcription polymerase chain reaction (qRT-PCR)

The total RNA was isolated using the TRIzol reagent (Invitrogen), and cDNA was synthetized using the PrimeScript™ Kit (TaKaRa Bio Inc., Dalian, China), following the manufacturer’s instructions. qRT-PCR was performed in triplicate using SYBR Green fluorescent-based assay (GeneCopoeia, Guangzhou, China) on a ViiATM6 RT-PCR system (Applied Biosystems, Carlsbad, CA). The primers used for real-time PCR are as follows: AC115619.1 forward: 5′-TGA​TGA​TAT​CGA​CGT​GAG​GTT​CC-3′, reverse: 5′-ATC​AAA​CAC​GTT​ATC​CTT​GAG​TCC-3’; GAPDH forward: 5′-CGG​AGT​CAA​CGG​ATT​TGG​TCG​TAT-3′, reverse: 5′-AGC​CTT​CTC​CAT​GGT​GGT​GAA​GAC-3’. Relative mRNA expression levels were calculated by the 2^−ΔΔCt^ method and were normalized to the internal control of GAPDH.

### 2.11 Immunohistochemistry

Tissues were fixed in 10% formalin, dehydrated using graded concentrations of ethanol, and embedded in paraffin. Then, 4-μm-thick sections were processed for analyses. Dewaxing, hydrating, and heat-mediated antigen retrieval with pH 9.0 Tris/EDTA buffer were performed. Subsequent antigen–antibody reactions and IHC staining were performed, according to the protocol of a commercial detection kit (ZSGB-BIO, Beijing, China). Antibodies for RBMX were diluted to recommended concentrations, following the manufacturer’s protocol (Abcam, Cambridge, United Kingdom). The immunoreactivity-tested protein was scored according to the percentage of positive staining cells and staining intensity, as described previously (Deng et al., 2020).

### 2.12 Cell culture and transfection

HCC cell lines SNU-449 and HepG2 were obtained from Procell Life Science and Technology (Wuhan, China) and cultured in RPMI-1640 or Dulbecco’s modified Eagle’s medium (DMEM) (Invitrogen, Carlsbad, CA) containing 10% fetal bovine serum (Procell Life Science and Technology). The cells were grown in a cell incubator with 5% CO_2_ at 37°C. Full-length AC115619.1 was amplified by PCR and cloned into the expression vector pcLV3 for AC115619.1 overexpression. The pcLV3–AC115619.1 plasmid or an empty vector was transfected into SNU-449 and HepG2 cells using the Lipofectamine 3000 reagent (Invitrogen). The cells were harvested 48 h after transfection for further analysis.

### 2.13 Cell viability assay

The cells were equivalently pipetted into a 96-well plate 48 h after transfection, and the cell viability was determined using the Cell Counting Kit-8 (CCK-8) (Dojindo Molecular Technologies, Inc., Tokyo, Japan) for different time points. Briefly, 10 μL of CCK-8 was added to each well, and the absorbance was measured at 450 nm.

### 2.14 5-Ethynyl-20-deoxyuridine (EdU) assay

We used an EdU kit (RiboBio, Guangzhou, China) to detect the proliferation ability of HCC cells. The cells were seeded and grown in a 96-well plate with a density of 5 × 10^3^ cells. Then, the cells were incubated with 50 μM EdU buffer at 37°C for 2 h, and then fixed and washed at room temperature. The cells were then permeabilized with 0.5% Triton X-100 for 10 min and subsequently reacted with the Apollo^®^ staining solution for 0.5 h in the dark. The cells were washed, and then, Hoechst 33342 was added to stain the nuclei. Images were visualized and captured using a fluorescence microscope.

### 2.15 Cell migration assay

Wound healing assay was used to detect the migration ability. Cells were seeded into 6-well plates after transfection with the plasmid for 48 h. Then, the confluent cell monolayers were scratched straightly using a 200-μL pipette tip. The cells were washed with PBS and cultured in fresh medium containing 1% FBS and 1% BSA for 72 h. Images of the scratched cells were captured using an inverted microscope.

### 2.16 Transwell invasion assay

Matrigel-coated upper inserts containing polycarbonate filters with a pore size of 8 μm (Corning, Tewksbury, MA) were used to assess the cell migration ability. The cells were suspended in 200 μL of serum-free DMEM or RPMI 1640 and cultured in the upper chambers and incubated at 37°C for 48 h, while the lower chambers were covered with DMEM or RPMI 1640 containing 10% FBS. The cells which penetrated the filter were fixed with methanol and then stained with 0.1% crystal violet hydrate solution. Images of the invaded cells were captured using an inverted microscope.

### 2.17 Statistical analysis

Statistical analysis was performed using SPSS software (version 21.0, SPSS Inc., Chicago, IL). The continuous variable data are presented as the means ± standard deviations (SDs). Median survival time, log-rank *p*-value, adjusted *p*-value, 95% confidence interval (CI), and hazard ratio (HR) were calculated using the Kaplan–Meier and Cox proportional hazard regression models. The χ^2^ test was performed to compare the categorical variables assessing the pathological and clinical characteristics. The differences between the experimental groups were analyzed using Student’s t-test or one-way ANOVA. *p* < 0.05 was considered to be statistically significant.

## 3 Results

### 3.1 Identification of DElncRNAs in HCC patients

**FIGURE 1:**
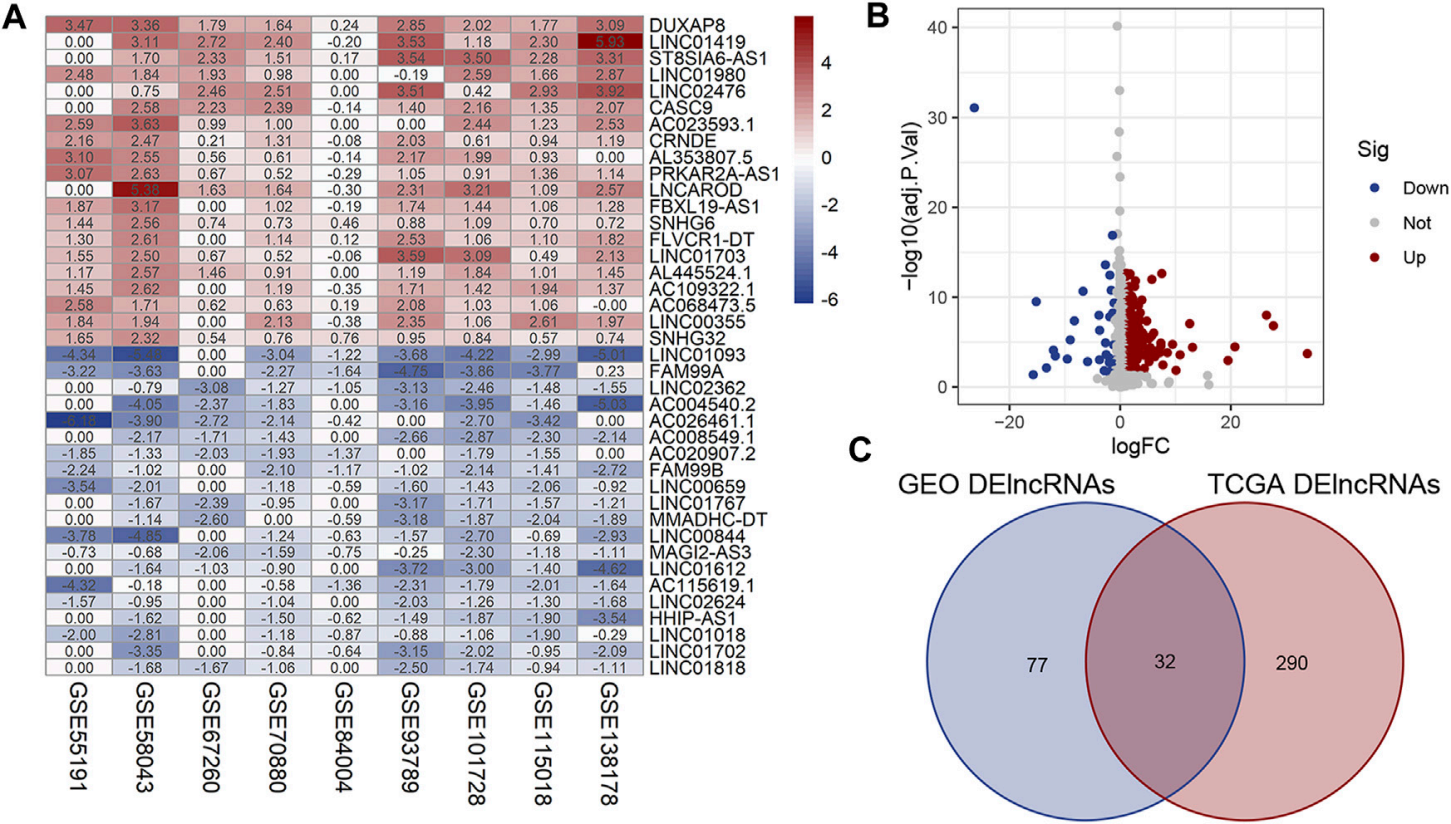
Identification of DElncRNAs in HCC. **(A)** Heatmap of DElncRNAs in HCC and normal liver tissues from the nine GSE microarrays. **(B)** Volcano plot for the DElncRNAs identified from TCGA dataset. **(C)** Intersection of DElncRNAs from GEO and TCGA databases. Row and column represent DElncRNAs/DEmRNAs and tissue samples, respectively. The color scale indicated the expression level of DElncRNAs. Red and blue represent up- and downregulation, respectively.

A total of 142 pairs of samples of HCC patients from nine GEO microarray datasets were enrolled to determine the expression level of lncRNAs in HCC tumor tissues and adjacent normal liver tissues. As shown in the heatmap given in Figure 1A, 51 upregulated lncRNAs and 58 downregulated lncRNAs in the GEO database were identified (|log_2_FC|>1, *p* < 0.05). Meanwhile, we identified 322 dysregulated lncRNAs (35 upregulation and 287 downregulation) in 374 HCC tumor samples and 50 normal liver tissues obtained from TCGA database (Figure 1B, |log_2_FC|>1, *p* < 0.05). DElncRNAs from the two platforms were then converged, and we finally obtained 32 lncRNAs which were significantly dysregulated in both GEO and TCGA databases (Figure 1C).

### 3.2 Prognostic analysis of DElncRNAs

**FIGURE 2:**
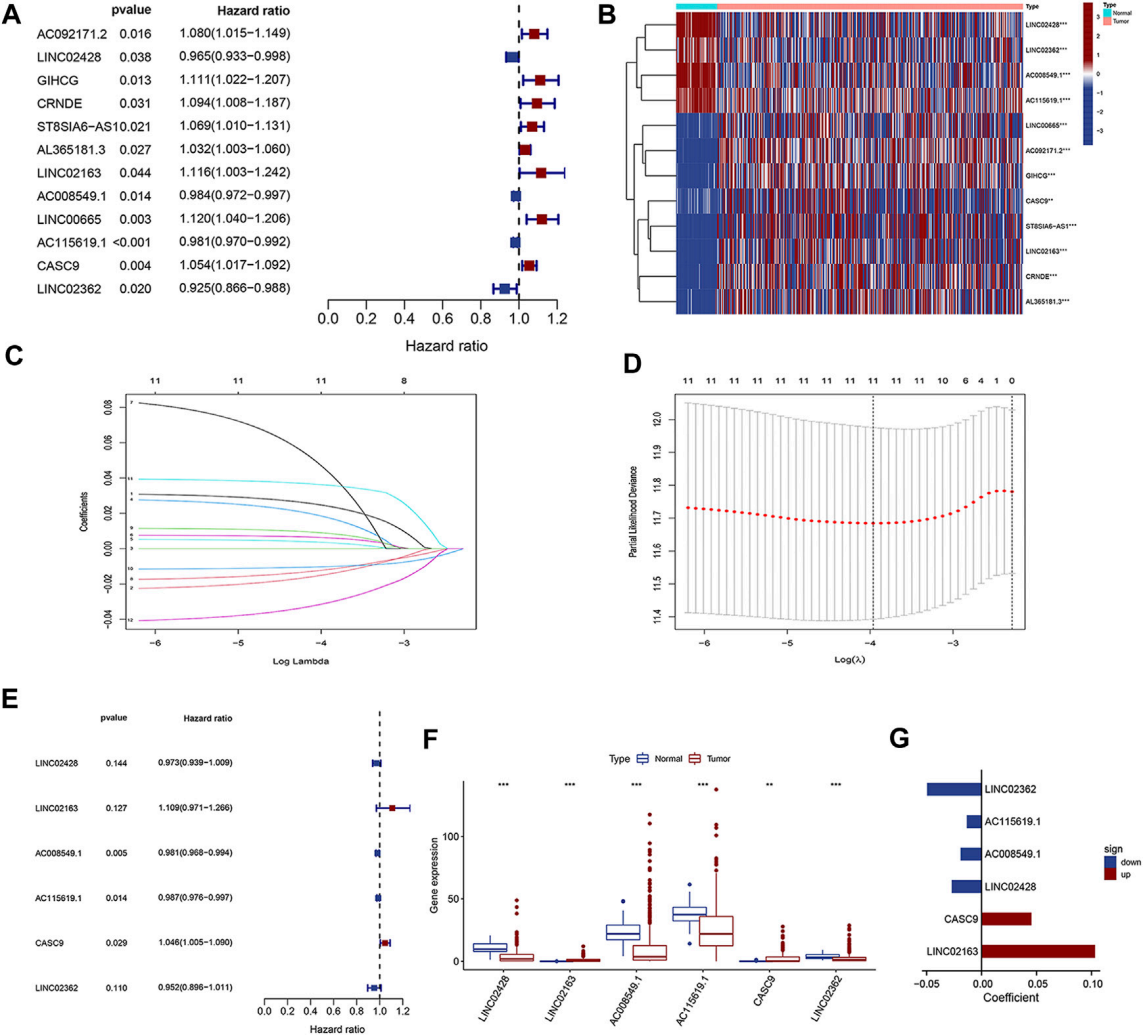
Identification of prognosis-related DElncRNAs. **(A)** Forest plot of univariate Cox regression analysis. **(B)** Heatmap of DElncRNAs from univariate Cox regression. **(C, D)** LASSO Cox analysis for DElncRNAs from univariate Cox regression. **(E)** Forest plot of multivariate Cox regression analysis. **(F)** Box plot and **(G)** forest plot for the six prognostic lncRNAs from multivariate Cox regression analysis.

Combining with the prognostic information, univariate Cox regression analysis was then performed to screen prognosis-related lncRNAs from the aforementioned 32 dysregulation lncRNAs in both datasets. Finally, 12 lncRNAs were found to be correlated with the prognosis of HCC patients in both datasets (Figure 2A, *p* < 0.05). As shown in the forest plot (Figure 2A), LINC02428, AC008549.1, AC115619.1, and LINC02362 were protective factors with HR < 1 in HCC patients, while AC092171.2, GIHCG, CRNDE, ST8SIA6-AS1, AL365181.3, LINC02163, LINC00665, and CASC9 were risk factors with HR > 1 (Figure 2A). The heatmap showed the altered expression of the aforementioned 12 prognosis-related lncRNAs in TCGA dataset (Figure 2B). We performed LASSO Cox analysis based on the 12 prognostic lncRNAs to identify the prognostic lncRNAs more accurately and filtered six key lncRNAs (i.e., LINC02428, LINC02163, AC008549.1, AC115619.1, CASC9, and LINC02362) (Figures 2C,D) using a dimensionality reduction method. Then, a multiple stepwise Cox regression analysis was further conducted to assess which lncRNA contributed most to the prognosis of HCC patients when combined together. The results showed that AC008549.1, AC115619.1, and CASC9 were suggested to be an independent prognostic factor of HCC patients (*p* < 0.05, Figure 2E). Expression levels of these lncRNAs in tumor tissues compared to normal tissues were also displayed (Figures 2F,G).

### 3.3 Establishment of a prognostic risk model

**FIGURE 3:**
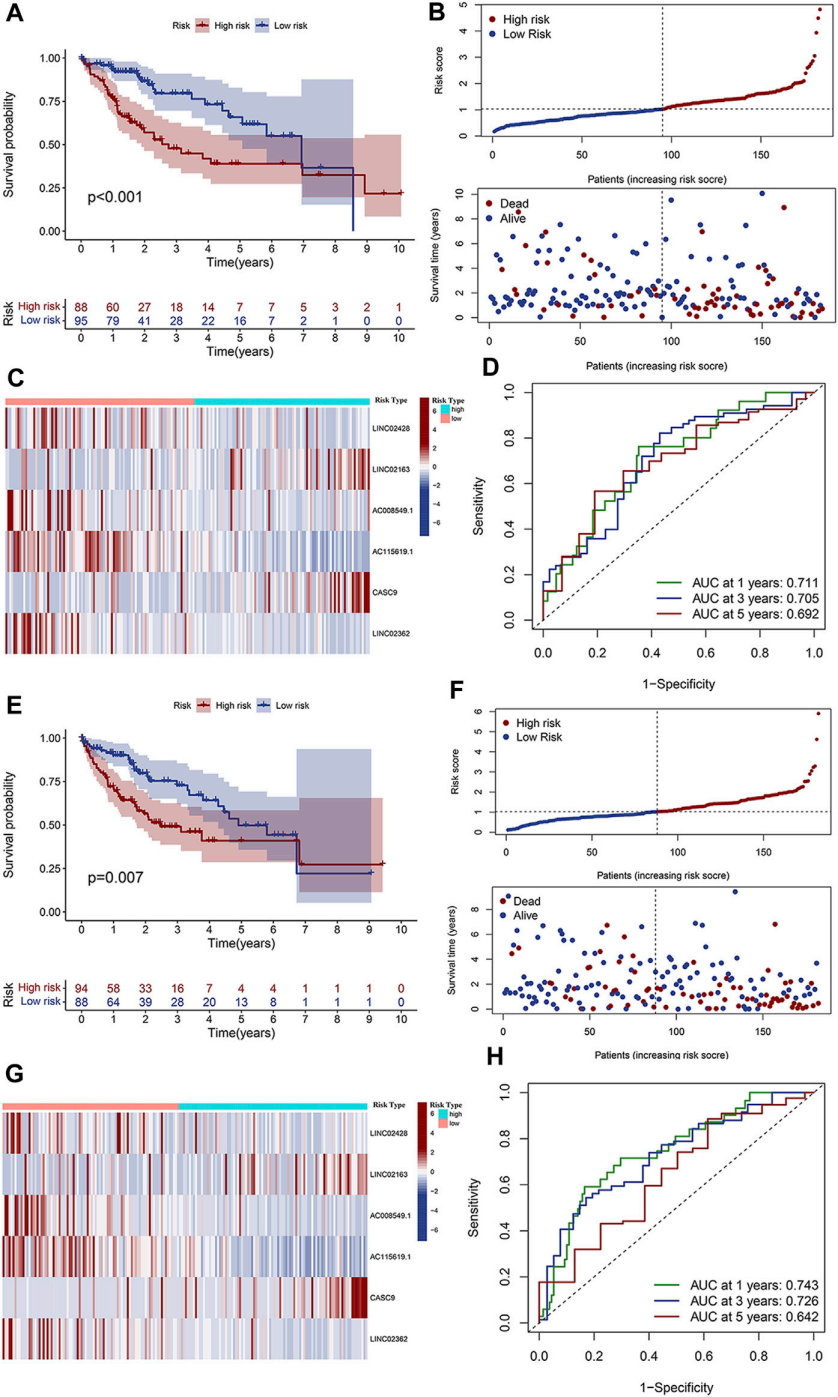
Validation of the clinical significance of the prognostic risk model. **(A)** Kaplan–Meier curves depicted the overall survival of patients in the training cohort from TCGA databases. Patients were divided into low- and high-risk groups based on the median value of the risk score. **(B)** Distributions of risk scores (upper) and survival status (lower) of HCC patients in the training cohort. **(C)** Expression heatmap of the six prognostic lncRNAs with low- and high-risk scores in the training cohort. **(D)** ROC curve of the prognostic signature for predicting the 1/3/5-year survival in the training cohort. **(E)** Overall survival curve of patients with low- and high-risk scores in the test cohort. **(F)** Distributions of risk scores (upper) and survival status (lower) of HCC patients in the test cohort. **(G)** Expression heatmap of the six prognostic lncRNAs with low- and high-risk scores in the test cohort. **(H)** ROC curve of the prognostic signature for predicting the 1/3/5-year survival in the test cohort.

**FIGURE 4:**
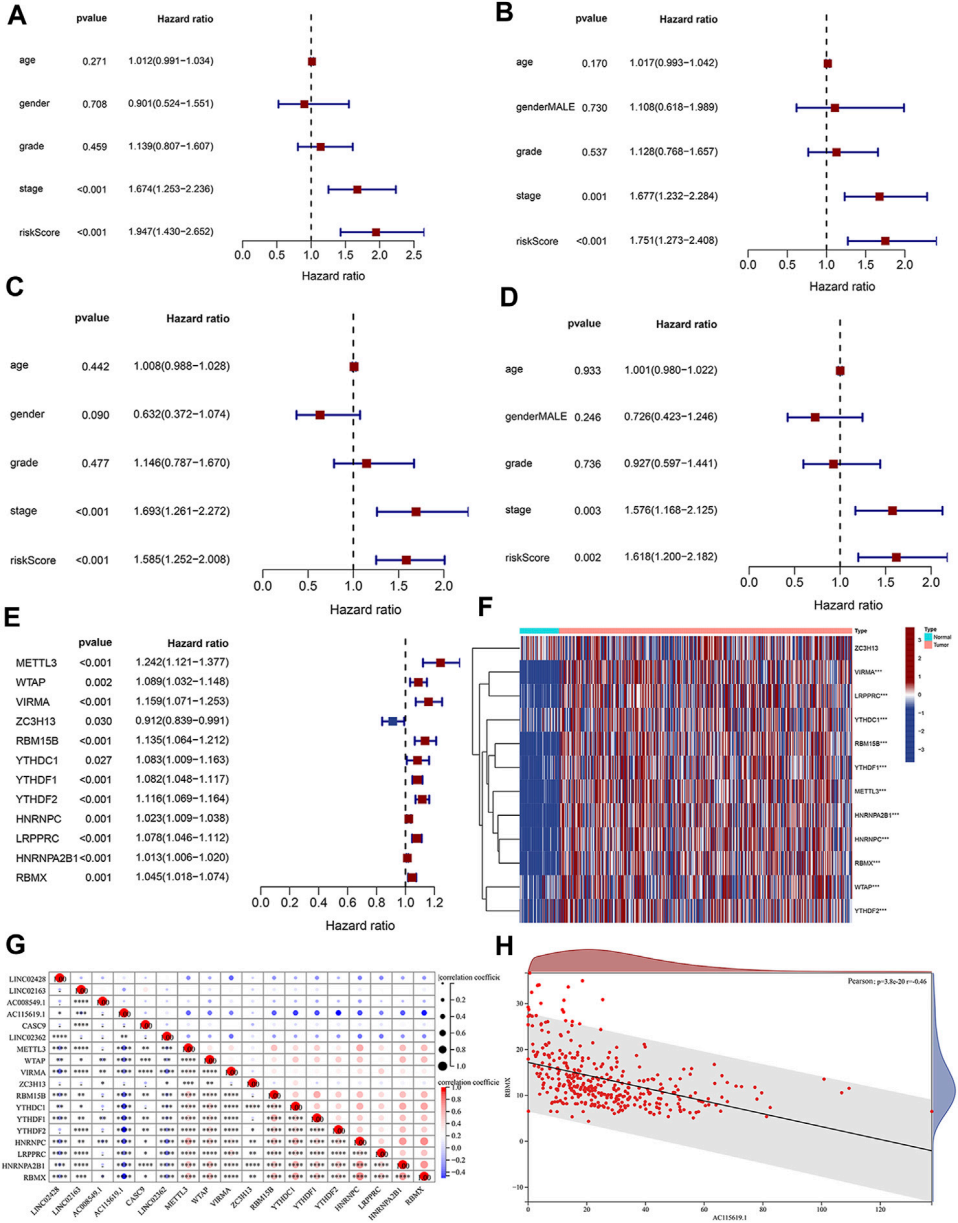
Clinical significance analysis of the risk score and the correlation of m6A and lncRNAs. Forest map of **(A)** univariate and **(B)** multivariate Cox regression analyses in the training cohort. Forest map of **(C)** univariate and **(D)** multivariate Cox regression analyses in the test cohort. **(E)** Univariate Cox regression analysis of the prognostic value of m6A-related regulators. **(F)** Heatmap of prognostic m6A regulators in tumor tissues and normal liver tissues. **(G)** Expression correlation between the six prognostic DElncRNAs and 12 m6A regulators. **(H)** Expression correlation between the lncRNA AC115619.1 and RBMX determined by Pearson’s coefficient analysis.

Based on each coefficient of the six prognostic lncRNAs in the multivariate Cox regression model, a risk score was calculated for each HCC patient in TCGA dataset. We then established a novel prognostic signature with the six lncRNAs. Patients in both training and test cohorts were divided into low- and high-risk subgroups, according to the median value of risk scores. Kaplan–Meier survival curves showed that HCC patients with high-risk scores had poor prognosis (Figures 3A,E). Survival status distributions suggested that patients in the high-risk score group suffered from higher mortality rates than low-risk score patients (Figures 3B,F). The heatmap revealed that the expression levels of prognosis-related lncRNAs were higher than those in patients with a low-risk score (Figures 3C,G). ROC curves were also performed to evaluate the predictive accuracy of this prognostic risk model, which demonstrated that prognostic lncRNAs harbored a potential ability to predict the OS (training cohort: 1-year AUC = 0.711 and test cohort: 1-year AUC = 0.743; Figures 3D,H). Univariate Cox regression was also utilized to analyze the prognostic value of the risk score and the patient’s clinical characteristics, including age, gender, grade, and stage. The results revealed that the stage and risk score of our model were positively correlated with poor prognosis in HCC patients (HR > 1, *p* < 0.05; Figures 4A,C). Moreover, multivariate Cox regression indicated that the stage and risk score were independent prognostic factors for HCC patients (*p* < 0.05; Figures 4B,D).

### 3.4 Correlation analysis of prognostic lncRNAs and m6A-related regulators

Emerging evidence has shown that RNA modification plays an important role in the expression and function of lncRNA. N6-methyladenosine (m6A) is the most abundant modification of RNA, and m6A regulators contribute to HCC by regulating various biological processes. To elucidate the relativity of m6A methylation and prognosis-related lncRNAs, we performed univariate Cox regression to screen the prognostic m6A regulators in HCC patients from TCGA dataset. The results showed that 12 m6A regulators were significantly correlated with the prognosis of HCC patients (Figure 4E, *p* < 0.05), and the heatmap depicted their expression alteration in tumor tissues and normal tissues (Figure 4F). Pearson’s correlation coefficient was further conducted to analyze the relationship between prognostic m6A-related regulators and six prognostic lncRNAs obtained from the prognostic signature (Figure 4G). The m6A-related regulator RBMX was found to be significantly correlated with the prognostic lncRNA AC115619.1 (Figure 4H; cor = −0.46, *p* = 3.8e-20).

### 3.5 Validation of the potential significance of the hub lncRNA AC115619.1 in public databases

**FIGURE 5:**
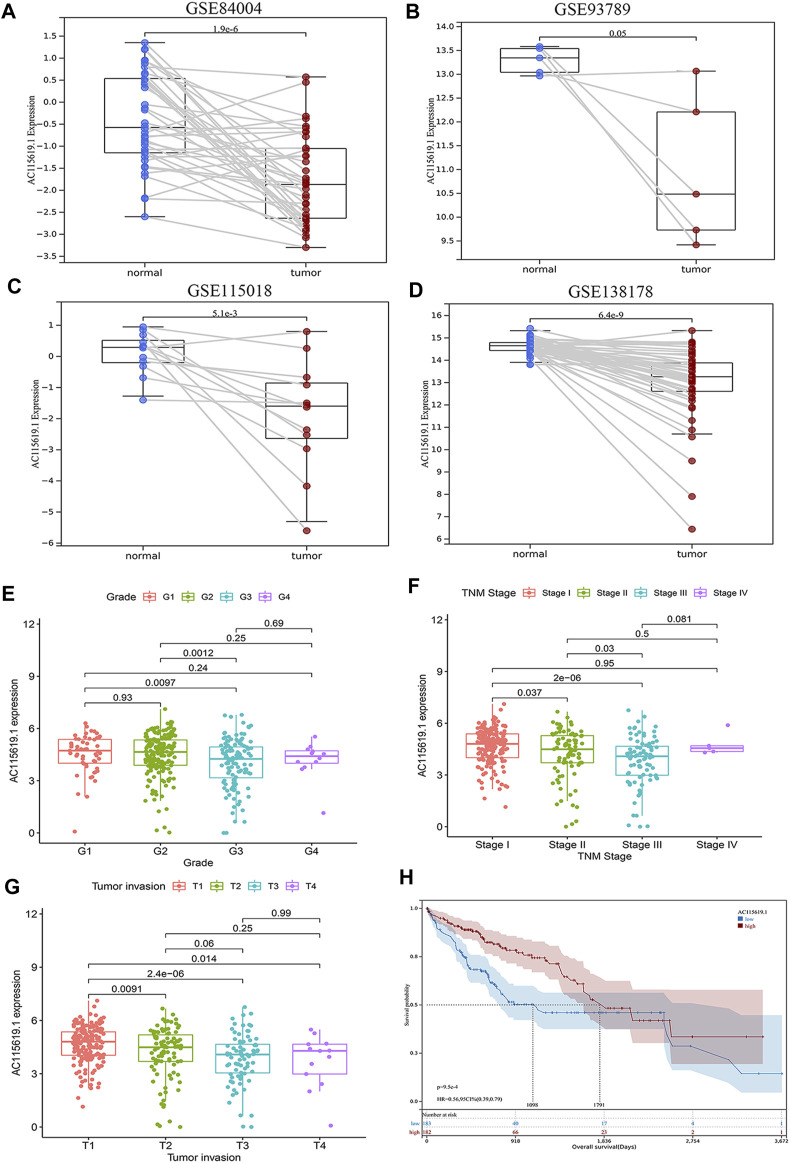
Validation of the expression and clinical value of lncRNA AC115619.1. **(A–D)** Validation of the low expression of lncRNA AC115619.1 in the public GSE microarray of the GEO database. **(E–G)** Correlations between AC115619.1 expression and tumor grade, stage, and invasion. **(H)** Overall survival Kaplan–Meier curves of patients with low and high expression of AC115619.1.

Since AC115619.1 has significantly contributed to HCC prognosis, is correlated with the m6A-related regulator RBMX tightly, and has never been reported in HCC, we selected AC115619.1 as a hub lncRNA for further investigation. Then, four datasets from the GEO database were used to validate the expression of lncRNA AC115619.1 in HCC patients. GSE84004, GSE93789, GSE115018, and GSE138178 datasets all showed that the expression of lncRNA AC115619.1 in HCC tumor tissues was significantly lower than that in normal tissues (Figures 5A–D; *p* < 0.05). We further analyzed the association between high and low AC115619.1 expression and the clinicopathologic characteristics of HCC patients obtained from TCGA database. As shown in Table 2, AC115619.1 expression was associated with tumor grade (*p* = 0.013), tumor invasion (*p* < 0.001), and TNM stage (*p* < 0.001). However, AC115619.1 expression was not associated with age (*p* = 0.714), gender (*p* = 0.765), lymph node metastasis (*p* = 0.974), and distant metastasis (*p* = 0.553, Table 2). We also verified the clinical significances in HCC patients with different subsets obtained from TCGA database. The expression of lncRNA AC115619.1 was found to be correlated negatively with tumor grade, tumor invasion, and, partly, TNM stage (Figures 5E–G). Additionally, the prognostic value of lncRNA AC115619.1 in predicting the patient’s OS was estimated, as shown in Figure 5H. The Kaplan–Meier curve showed that HCC patients with high AC115619.1 expression had a better OS obviously (Figure 5H; *p* < 0.05; HR = 0.56, 95% CI: 0.39–0.79). These data indicated a tumor suppressor role of AC115619.1 in HCC.

**TABLE 2:** Correlation of AC115619.1 expression in HCC tissues with patients’ clinicopathologic features from TCGA dataset.

Clinicopathologic variable		AC115619.1 expression (n = 363)		
		Low	High	*p*-value
Age (years)	≤61	95	91	0.714
	>61	87	90	
Gender	Men	121	123	0.765
	Women	61	58	
Grade	G1∼2	105	125	0.013
	G3∼4	76	52	
Tumor invasion	T1∼2	119	151	<0.001
	T3∼4	63	27	
Lymph node metastasis	No	125	121	0.974
	Yes	2	2	
Distant metastasis	No	129	132	0.553
	Yes	2	1	
TNM stage	I ∼ II	111	142	<0.001
	III ∼ IV	58	28	

### 3.6 Construction of a ceRNA network and functional enrichment analysis

**FIGURE 6:**
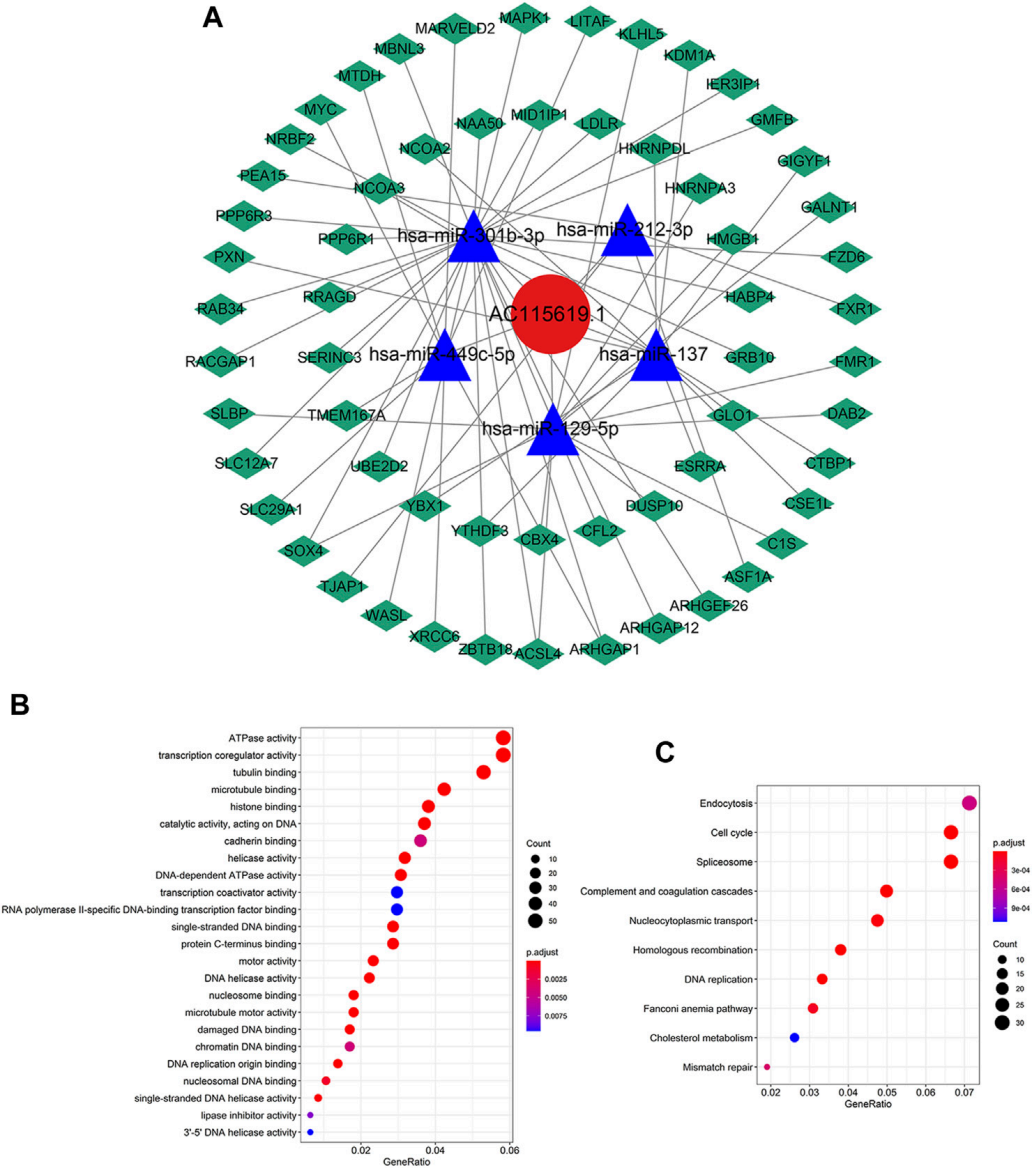
Functional enrichment analysis of AC115619.1. **(A)** ceRNA network of AC115619.1 (red), its target miRNAs (blue), and corresponding target mRNAs (green). **(B)** Gene Ontology (GO) enrichment and **(C)** KEGG pathway analyses of AC115619.1. Dot size represents the count of relative genes, and the color represents the p-value.

We also constructed a ceRNA network through the miRcode database to explore the potential interaction miRNAs of AC115619.1. We found that there were 11 miRNAs that possessed interaction positions with lncRNA AC115619.1 (Supplementary Figure S1A). The target mRNAs of miRNAs which potentially interact with AC115619.1 were further screened by combining the miRDB, miRTarBase, and TargetScan databases together. For predicting the target mRNAs more accurately, these screened mRNAs were further intersected with HCC differentially expressed mRNAs (DEmRNAs) obtained from TCGA database. A total of five miRNAs, namely, miR-212-3p, miR-129-5p, miR-301b-3p, miR-449c-5p, and miR-137, which might regulate the 60 DEmRNAs, were finally identified (Figure 6A, |log_2_FC|>2, *p* < 0.01). To further investigate the biological insights and pathway of lncRNA AC115619.1, we performed GO and KEGG analyses. GO annotation revealed that the biological processes of AC115619.1 were primarily associated with ATPase activity, transcription co-regulator activity, and tubulin binding (Figure 6B). The KEGG pathway analysis showed that AC115619.1 was involved in the pathway of endocytosis, cell cycle, and spliceosome (Figure 6C). Additionally, genes/enzymes from the most remarkable enrichment cell cycle pathway were negatively correlated with the expression of AC115619.1 in HCC (Supplementary Figure S1B).

### 3.7 Patient responses to chemotherapy and targeted therapy, and the immunocyte infiltration landscape of AC115619.1

**FIGURE 7:**
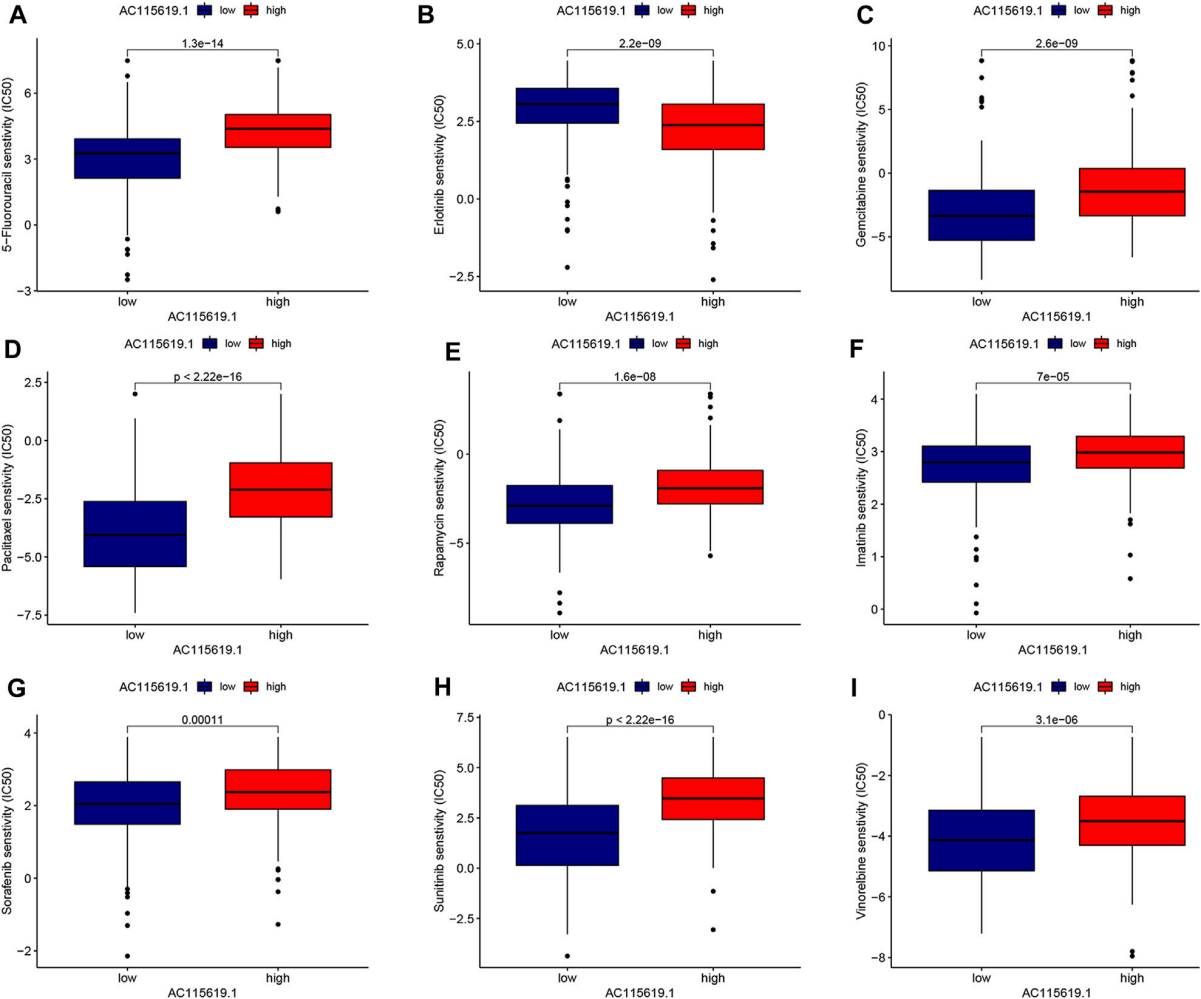
Prediction for the drug IC_50_ value of AC115619.1 in HCC. **(A–I)** Differential IC_50_ values of chemotherapy and targeted therapy responses in high and low expression of AC115619.1. The high expression of AC115619.1 was related to a higher IC_50_ value for most chemotherapy and targeted drugs.

**FIGURE 8:**
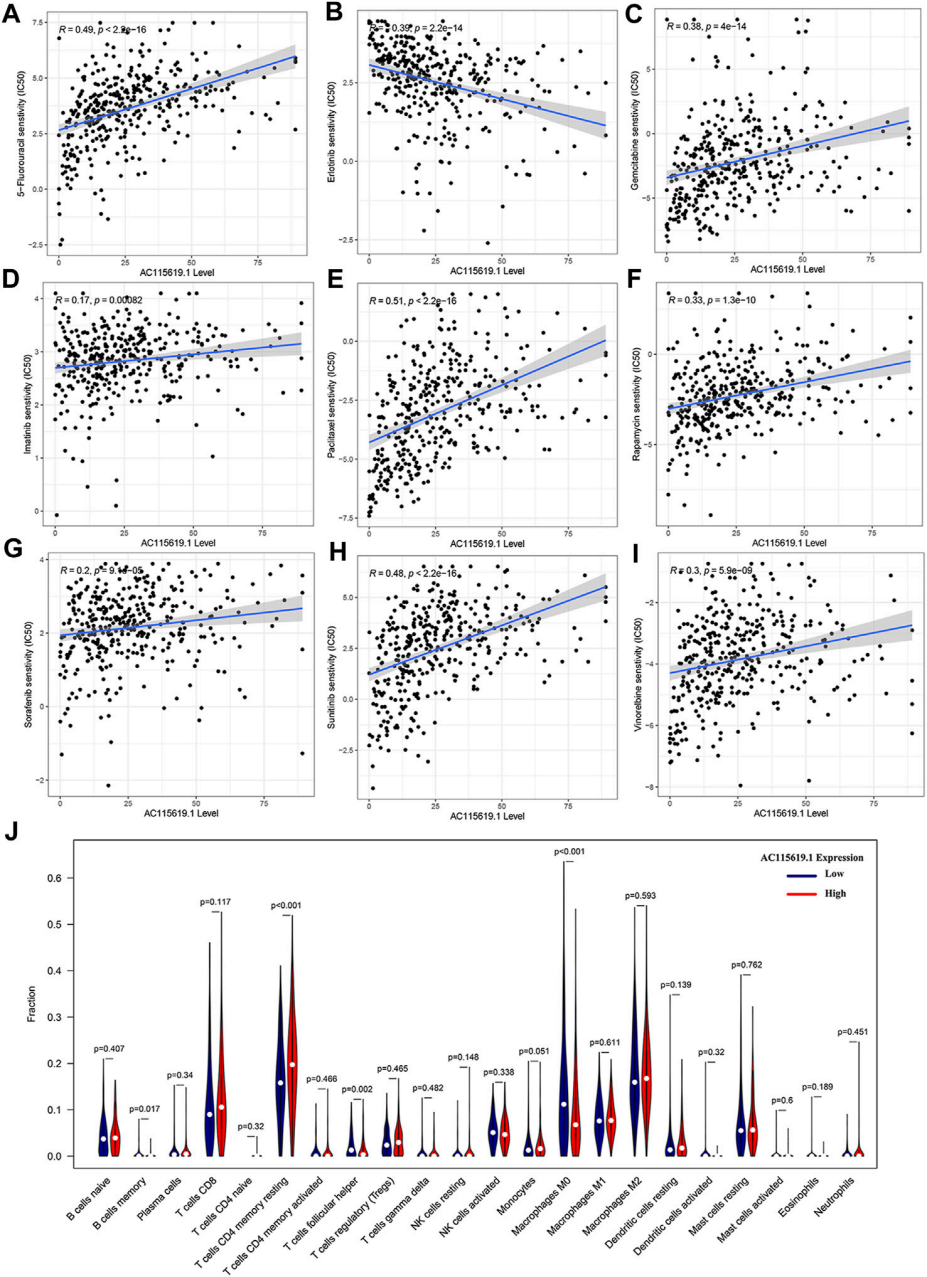
Drug sensitivity and immunocyte infiltration analysis of AC115619.1. **(A–I)** Detailed correlation of drug sensitivity and the expression of AC115619.1. **(J)** Box plot of 22 hematopoietic cell phenotypes between differential expression of AC115619.1.

To promote the potential clinical application, we predicted the IC_50_ value of commonly used chemotherapeutic and targeted agents in high and low AC115619.1 expression groups based on the algorithm provided in the pRRophetic R package. The IC_50_ values of 5-fluorouracil, gemcitabine, paclitaxel, rapamycin, imatinib, sorafenib, sunitinib, and vinorelbine were higher in the high AC115619.1 expression group of HCC patients, indicating that HCC patients with low AC115619.1 expression were more sensitive to these eight drugs (Figures 7A–I). The detailed correlation was also provided (Figures 8A–I). Emerging evidence indicates that the immune microenvironment plays an important role in tumor progression. We also investigated the tumor immunocyte infiltration proportion between high and low AC115619.1 expression patients using the CIBERSORT algorithm. The results showed that memory B cells (*p* = 0.017), CD4 memory resting T cells (*p* < 0.001), T follicular helper cells (*p* = 0.002), and M0 macrophages (*p* < 0.001) were significantly enriched in the high and low AC115619.1 subgroups (Figure 8J).

### 3.8 Validation of the lncRNA AC115619.1 and RBMX in the local cohort

**FIGURE 9:**
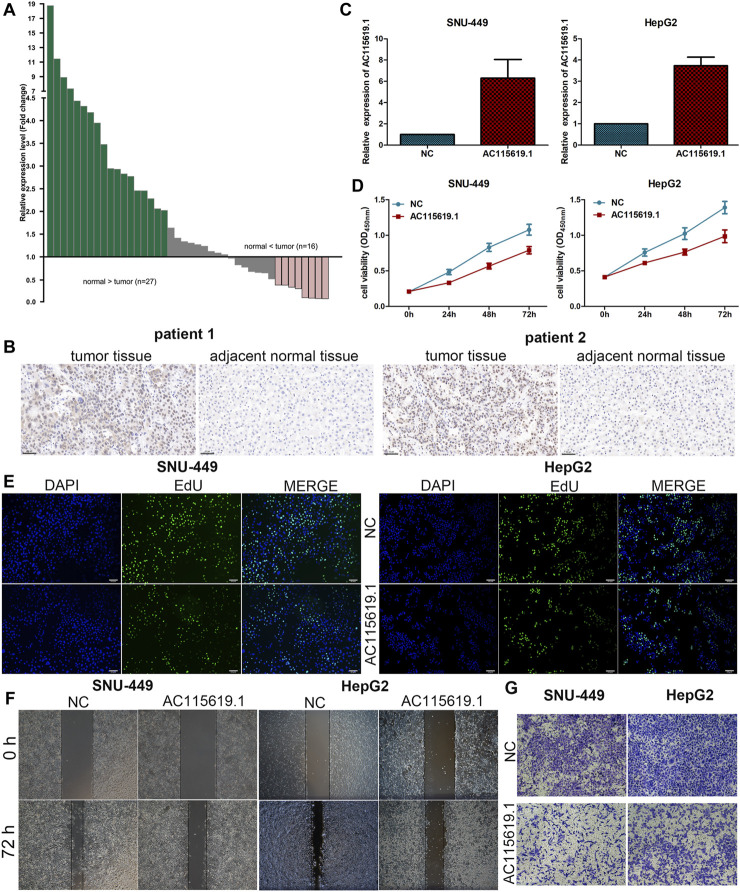
AC115619.1 was negatively related to RBMX and inhibited the progression of HCC. **(A)** Downregulation of AC115619.1 was validated by qRT-PCR in local HCC samples. Fold changes are analyzed using the formula, 2^- (△△CT (tumor/adjacent normal tissue))^. **(B)** Representative IHC pictures of RBMX expression in HCC tumor and adjacent normal liver tissues (original magnification: ×200). **(C)** Transfected efficiency of the AC115619.1 plasmid in HCC cells detected by qRT-PCR. **(D)** Cell viability of HCC cells after AC115619.1 overexpression detected by CCK8 assay. **(E)** EdU assay displayed the proliferating ability of HCC cells. **(F)** Migration ability determined by wound healing assay. **(G)** Invasion ability determined by transwell assay.

To validate the expression of AC115619.1 in local samples, we detected its expression level in our collected 43 pairs of HCC samples using the qRT-PCR assay. Our results demonstrated that AC115619.1 was downregulated in most HCC tumor samples compared to adjacent normal tissues (Figure 9A). Among the 43 pairs of HCC patient samples, the expression of AC115619.1 in 27 normal liver tissues was higher than that in HCC samples (Figure 9A). To further evaluate the correlation between AC115619.1 and m6A-related regulator RBMX, immunohistochemistry staining was performed in these 43 pairs of HCC samples. As shown in Figure 9B, typical pictures of IHC staining revealed that RBMX was localized in the cell nucleus and the expression of RBMX in tumor tissues was higher than that in adjacent normal liver tissues, which was negatively correlated with the AC115619.1 expression in HCC (Figure 9B).

### 3.9 Overexpression of AC115619.1 inhibited the proliferation, migration, and invasion of HCC cells

Since AC115619.1 expression was downregulated in HCC tissues and low AC115619.1 expression is closely related to a poor prognosis of HCC patients, we used SNU-449 and HepG2 cell lines for further experiments. After transfection with the plasmid, the expression level of AC115619.1 was significantly upregulated, as confirmed by qRT-PCR (Figure 9C). CCK-8 and EdU assays demonstrated that overexpression of AC115619.1 inhibited the proliferation of HCC cells (Figures 9D,E). In addition, the wound healing migration and transwell invasion experiments revealed that overexpression of AC115619.1 repressed the migration and invasion abilities in both SNU-449 and HepG2 cells (Figures 9F,G). Taken together, our results suggested that AC115619.1 inhibited the progression of HCC.

## 4 Discussion

HCC is characterized with a low diagnosis rate and rapid progression at the early stage. Most patients are diagnosed at an advanced stage and lost the opportunity to receive curative surgery treatment. Due to the insidiousness and heterogeneity of HCC, there is no appropriate biomarker to accurately predict clinical prognosis. Therefore, developing novel biomarkers is important to improve the clinical outcomes of HCC patients.

In recent years, many biomarkers have been identified, owing to the great development of microarray and high-throughput sequencing technologies. LncRNAs have been investigated and proposed to be potential diagnostic and therapeutic biomarkers in human malignancies (Iaccarino and Klapper, 2021). In HCC, the aberrant expression of lncRNAs has been reported to be a potential biomarker for early diagnosis and predicting the prognosis (Dickson, 2016; Huang et al., 2020). For instance, lncRNA-D16366 was found to be downregulated in both tumor tissues and serum samples of HCC patients, which implied a significant value of diagnostic and prognosis prediction with its aberrant expression (Liu et al., 2014; Chao and Zhou, 2019). The lncRNA AC099850.3 was reported to be overexpressed and accurately predicted the prognostic outcomes of HCC patients (Wang et al., 2022). The present study identified 32 HCC-related DElncRNAs overlapping from 516 HCC patients obtained from GEO and TCGA datasets. Six lncRNAs (LINC02428, LINC02163, AC008549.1, AC115619.1, CASC9, and LINC02362) were screened by univariate Cox and LASSO regression and were then selected to construct a novel prognostic signature. Our results showed that patients with a high-risk score in the prognostic signature had a better survival rate, and the risk score was an independent prognostic factor for HCC patients in both training and test cohorts. This might provide a sensitive and specific model in predicting the patient’s outcomes. Among the six lncRNAs in the prognostic model, LINC02163 and CASC9 were found to be upregulated in various cancers including HCC. LINC02163 and CASC9 were closely associated with the patient’s survival and acted as a candidate prognostic biomarker with their significant values (Dong et al., 2018; Qin et al., 2020; Qi et al., 2021; Tian et al., 2021). LINC02362 and AC008549.1 were identified to be tumor-inhibitory lncRNAs and contributed to the patient’s survival of HCC, while the lncRNA AC008549.1 was classified as a pyroptosis-related signature (Wang et al., 2022; Li et al., 2022). AC115619.1 was reported to be a ferroptosis-related lncRNA and showed to be an independent prognostic factor in gastric adenocarcinoma (Fu et al., 2020; Cai et al., 2022). Consistently, our results supported the aberrant expressions of the six lncRNAs in previous reports. Our multivariate Cox regression identified three lncRNAs (AC008549.1, AC115619.1, and CASC9) to be independent factors in HCC.

Since there is no report of AC115619.1 in HCC yet, we selected it as a hub lncRNA for further exploration. The clinical significance and expression level of AC115619.1 were validated both in public datasets and local HCC samples. To analyze its targeted miRNAs and potential pathways, a suite of bioinformatics methods was executed subsequently. Meanwhile, we analyzed the correlation between the differential expression of AC115619.1 and immune cell infiltration. The drug sensitivity of AC115619.1 was also provided to predict the IC_50_ value of chemotherapeutic and targeted agents for each patient. The results showed that the high expression of AC115619.1 had higher IC_50_ values in most common drugs used in HCC. In addition, we explored and validated an inhibitory biological function of AC115619.1 in HCC cells. Overexpression of AC115619.1 by the plasmid inhibited the proliferation, migration, and invasion *in vitro*. Combined with the aforementioned results, AC115619.1 exerted its potential therapeutic value by serving as an independent prognostic factor and tumor suppressor in HCC.

N^6^-Methyladenosine (m6A), the most popular and common modification of mRNA, exerts a tremendous effect on posttranscriptional regulation (Zhang et al., 2020). In recent years, evidence revealed that m6A modification exists on non-coding RNA, including lncRNAs, and plays a critical role in deciding the fate of lncRNAs (Chen et al., 2020). The regulation effects of m6A modification might be attributed to the m6A regulators, which include methyltransferases, demethylases, and binding proteins. M6A regulators have been found to be the key component of m6A modification and play an essential role in the progression of human malignancies (Wang et al., 2017; Gao et al., 2021). It is reported that overexpression of METTL3 (m6A writer) increased the m6A level of colon cancer by enhancing the expression and protein binding effect of lncRNA RP11. As a result, METTL3 promoted the metastasis of colon cancer (Wu et al., 2019). The abnormal increase in m6A regulators has been revealed to be involved in the progression, drug sensitivity, and immune response of HCC, suggesting that targeting m6A-modified lncRNAs might be a potential therapy strategy for HCC (Chen et al., 2020; Lin et al., 2020). METTL3 upregulated the expression of LINC00958 and LNCAROD to regulate the malignant phenotype of HCC (Zuo et al., 2020; Jia et al., 2021). KIAA1429, a component of the m6A methyltransferase complex, promoted the growth and metastasis of HCC by mediating m6A modification on GATA3 pre-mRNA (Lan et al., 2019). Herein, we identified 12 m6A regulators which were significantly related to the prognosis of HCC patients by univariate Cox regression analysis. Then, we revealed the correlation between the 12 m6A regulators and the six lncRNAs in the prognostic model and found that RBMX was the most correlated with the prognostic lncRNA AC115619.1. RBMX has been reported to be overexpressed in HCC tissues and cell lines, favoring malignant behavior and sorafenib resistance of HCC (Song et al., 2020). Similarly, our analysis showed that RBMX was highly expressed in HCC tumor tissues and negatively associated with the expression of lncRNA AC115619.1. Consistently, our results from the local cohort also confirmed the high expression of RBMX and the negative association with AC115619.1. Then, we hypothesized that the abnormal expression of lncRNA AC115619.1 might result from an m6A modification pattern through RBMX. However, the detailed relationship and whether m6A modification affects AC115619.1 need to be explored in the future.

In conclusion, this study identified and established a risk signature of HCC-related lncRNAs systematically, which could be applied to predict the prognosis of HCC patients. A novel lncRNA AC115619.1 was first identified as an independent prognostic factor for HCC patients, revealing an inhibitory effect of AC115619.1 and its correlation with RBMX. Our comprehensive evaluation of lncRNA provides a new therapeutic strategy for HCC patients.

## Data Availability

The datasets presented in this study can be found in online repositories. The names of the repository/repositories and accession number(s) can be found in the article/Supplementary Material.
