# Supplementary figures and images for: Identification of a novel lncRNA prognostic signature and analysis of functional lncRNA AC115619.1 in hepatocellular carcinoma
Source: Front Pharmacol. 2023 Aug 8;14:1167418. doi: 10.3389/fphar.2023.1167418 (PMC10442647; doi:10.3389/fphar.2023.1167418)

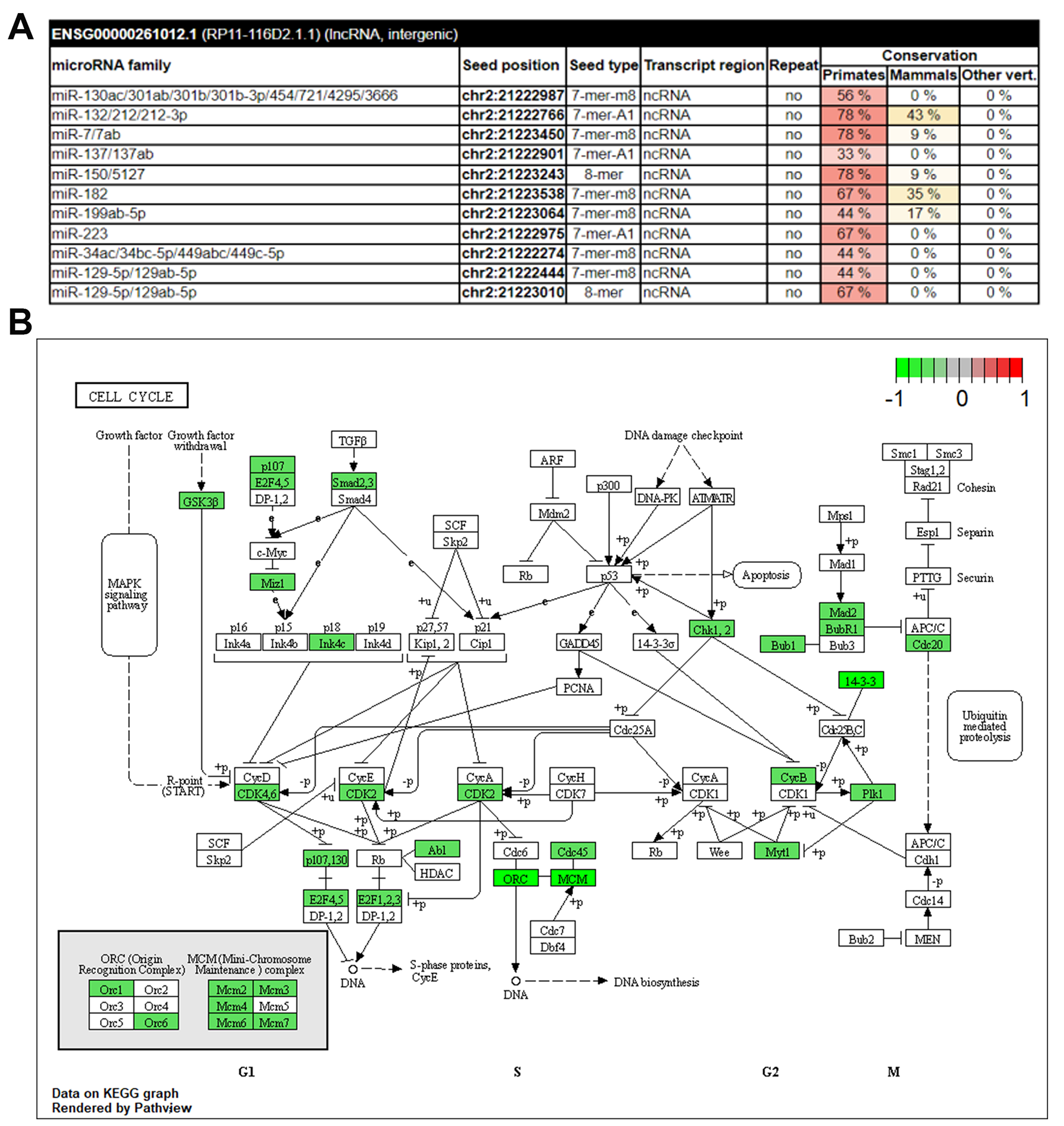

Supplement: Supplementary file 1 [file Image1.TIF]
